# Changes in glycaemic control of oral anti-diabetic medications assessed by continuous glucose monitors among patients with type 2 diabetes: a protocol of network meta-analysis

**DOI:** 10.1186/s13643-022-01986-5

**Published:** 2022-06-02

**Authors:** Mingyue Zheng, Adeel Khoja, Anamica Patel, Yunting Luo, Qian He, Xuan Zhao, Shenqiao Yang, Peng Hu, Wei Lin

**Affiliations:** 1 School of Health and Rehabilitation, Chengdu University of Traditional Chinese Medicine, Chengdu, 611137 China; 2 Adelaide Medical School, University of Adelaide, Adelaide, SA 5005 Australia; 3 Observatory Evidence Service, Public Health Wales, Cardiff, CF10 4BZ UK; 4 Center of Infectious Diseases, West China Hospital, Sichuan University, Chengdu, 610041 China; 5 Institute for Disaster Management and Reconstruction, Sichuan University, Chengdu, 610207 China; 6 Oftendon Trauma, No. 1 Orthopedics Hospital of Chengdu, Chengdu, 610000 China; 7 School of Pharmacy, Chengdu University of Traditional Chinese Medicine, Chengdu, 611137 China; 8 School of Management, Chengdu University of Traditional Chinese Medicine, Chengdu, 611137 China

**Keywords:** Oral anti-diabetic medications, Continuous glucose monitor, Health technology assessment, Diabetes management, Systematic review

## Abstract

**Background:**

Continuous glucose monitors (CGMs) can measure interstitial fluid glucose levels to provide comprehensive real-time glucose profile among people with type 2 diabetes. These can accurately detect glucose levels, hyperglycaemia and hypoglycaemia events compared with conventional self-monitoring. Increased application of CGMs provides a valuable opportunity to evaluate glucose control on oral anti-diabetic medications. This review will compare the efficacy and safety of oral anti-diabetic medications among patients with type 2 diabetes, evaluated by CGM.

**Methods:**

The following databases will be searched: Cochrane Library, PubMed, EMBASE, CINAHL, PsycINFO, Scopus and grey literature (ClinicalTrials.gov, PsycEXTRA, ProQuest Dissertations, Google Scholar and Theses Global) for the identification of studies. The review will include and summarise evidence from randomised clinical trials that use CGMs for blood glucose management in adults (aged ≥ 18 years), published in English between January 2000 and May 2021 without any restrictions of countries. Reference list of all selected articles will independently be screened to identify additional studies left out in the initial search. Primary outcomes will be HbA1c (≤ 7.0%), time spent with hypoglycaemia (< 70 mg/dl) or hyperglycaemia (≥ 180 mg/dl). Secondary outcomes will be change in weight, blood pressure and related comorbidities (cardiovascular mortality, heart failure events, myocardial infarction and stroke). Study selection, data extraction and quality assessment will be conducted independently by at least two reviewers. A third reviewer will determine and resolve discrepancies. At least two independent reviewers will cross-check data synthesis. The quality of evidence of the review will be assessed according to the Grading of Recommendations Assessment, Development and Evaluation (GRADE) Tool.

**Discussion:**

The review is anticipated to provide up to date evidence for further studies and clinic practices regarding glycaemic control, hypoglycaemia, and hyperglycaemia issues. The results will be published in a peer-reviewed journal.

**Trial registration:**

PROSPERO CRD42020188399.

**Supplementary Information:**

The online version contains supplementary material available at 10.1186/s13643-022-01986-5.

## Background

Diabetes is a progressive and chronic disease characterised by elevated blood glucose levels. Globally, the total number of patients with diabetes (aged 20–79 years) is expected to increase to 642 million by 2040 [1]. Around 95% of patients with diabetes have type 2 diabetes mellitus (T2D), and the majority of patients with T2D have to take medications lifelong [2]. Blood glucose control is the most crucial part of diabetes management, and lifestyle modification and pharmacological therapy are suggested for diabetes treatment and should be reviewed every three months [1, 3].

Existing guidelines and evidence recommend metformin as first-line treatment, sulfonylurea as second-line treatment (or first-line treatment if metformin is contraindicated) and insulin as third-line treatment [3–5]. Increasing evidence have suggested that anti-diabetic medications (ADMs) reduce the incidence of long-term complications, hospital admission, and mortality among patients with diabetes [6]. Evaluating the efficacy of short and long-term glycaemic changes of ADM have long been debated along with the increase demand of patient-centered diabetes treatment. For example, dipeptidyl peptidase-4 (DPP-4) inhibitors and sodium-glucose co-transporter 2 (SGLT-2) inhibitors have a low risk of hypoglycaemia; in contrary, sulfonylureas and thiazolidinediones may cause weight gain [3]. For example, hypoglycaemic events lead to an increased risk of cardiovascular disease and mortality in patients with T2D [7]. Therefore, it is crucial to evaluate existing evidence of the drug effects in terms of better drug selection and diabetes control.

CGM has become a useful assessment tool for real-time glucose monitoring in clinical and public diabetes management settings. It has been used to accurately detect hyperglycaemia and hypoglycaemia since 2000 [8–10]. Many randomised controlled trials (RCTs) [4, 11, 12] have used this new technology to monitor blood glucose control and side effects with oral ADM [i.e., metformin, sulfonylurea, SGLT-2 inhibitors, glucagon-like peptide 1 (GLP-1) agonists, and DPP-4 inhibitors] among adults with T2D. Compared with fingertip tests, using CGM can more accurately monitor the real-time glucose changes, reflect glycaemic changes and the effectiveness of traditional and new drugs.

The measurement of hypoglycaemia with the conventional fingertip blood glucose measurement method has a large error and is often ignored. With the development of science and technology, CGMs have presented excellent abilities in glucose management because of their accuracy and professionalism. Especially in the past decade, many RCTs have used CGM as an assessment tool to monitor real-time glycaemic changes of oral ADM with excellent feasibility and precision [11, 13, 14]. Therefore, it is necessary to perform a network meta-analysis (NMA) of those RCTs based on their results reported by CGMs. This evaluation tool can measure all index fluctuations in real-time and provide more detailed evidence of glycaemic changes.

Regarding glucose reduction, an NMA of 29 RCTs published in 2012 found that biphasic insulin, GLP-1 analogues and basal insulin were classified as the top 3 drugs of HbA1c reduction [15]. However, another NMA of 301 clinical trials found that there were no significant differences in the associations between any of nine available classes of ADM alone or in combination (metformin, sulfonylurea, thiazolidinediones, DPP-4 inhibitor, SGLT-2 inhibitor, basal insulin, basal-bolus insulin, α-glucosidase inhibitor, GLP-1 receptor agonist) and the risk of cardiovascular or all-cause mortality among patients with T2D [12].

However, the previous NMA of drug effectiveness of glucose control did not consider the impact of using a different assessment tool. In this sense, the implementation of CGM is an objective and sensitive assessment tool, if the included studies used traditional fingertips to measure blood glucose, it is easy to ignore the fluctuations of hypoglycaemia and hyperglycaemia. The use of different assessment tools would be an issue of heterogeneity if the included studies compared the outcomes of glucose levels collected by different methods (GCM detected or self-reported relevant event). Therefore, it is necessary to perform an NMA of oral ADM assessed by CGMs. This evaluation tool can measure all index fluctuations in real-time and provide more advance evidence. In practice, clinicians can choose various drugs based on guidelines and specific situations, but it is crucial to provide evidence generated by CGMs to make an optimal choice of glucose control.

Therefore, this review aimed to conduct an NMA to build a comprehensive profile of glycaemic control by different oral ADM based on CGM assessments and reports, especially the management of hypoglycaemic and hyperglycaemic symptoms, to provide a reference for T2D management.

## Methods

### Research aim and design

This review aimed to compare the efficacy and safety of oral anti-diabetic medications among patients with T2D, evaluated by CGM. The protocol has been registered in the International Prospective Register of Systematic Reviews (PROSPERO) CRD42020188399. This protocol was developed based on the Preferred Reporting Items for Systematic Review Protocols (PRISMA-P) Statement Preferred Reporting Items for Systematic Review and Meta-Analysis Protocols (Table S1) [16]. The PRISMA-NMA will be used to further direct the systematic review [17]. Additionally, this protocol will guide the actual systematic review and NMA. Any deviations while conducting this review will be stated including the reasons for the changes made in the methods of the final published systematic review.

### Eligibility criteria

This review has been designed according to the Population-Intervention-Comparators-Outcomes-Study design (PICOS) framework. Overall, RCTs involving adults with diagnosed T2D on any oral ADM as the intervention group and placebo/routine care as the control group, and having outcomes assessed by CGM will be part of this review. Studies comparing different types of ADM, assessed by CGM will also be included.

Inclusion criteria are as follows:

- Adults diagnosed with T2D (diagnostic criteria including the American Diabetes Association or World Health Organization or national guidelines); and
- T2D of at least 8 weeks duration

Exclusion criteria are as follows:

- Adults aged < 18 years;
- Other types of diabetes (i.e., gestational diabetes mellitus or idiopathic diabetes or type 1 diabetes);
- Patients in hospital or intensive care unit associated with serious conditions; or
- Non-RCT studies, non-T2D, follow-up duration less than eight weeks, conference abstracts and duplicate studies.

The intervention group was defined as patients who were taking the following oral ADMs according to the category (A10B) of ADMs on the Australian Pharmaceutical Benefits Scheme [18] and other Food and Drug Administration (FDA) approved oral ADMs. Traditional classes: oral insulin, biguanides (e.g. metformin), sulfonylureas (e.g. glibenclamide, gliclazide, glimepiride and glipizide); New classes: Alpha glucosidase inhibitors (e.g. acarbose), thiazolidinediones (e.g. pioglitazone), DPP-4 inhibitors (e.g. alogliptin, linagliptin, saxagliptin, sitagliptin and vildagliptin), GLP-1 analogues (e.g. dulaglutide, exenatide and semaglutide), SGLT-2 inhibitors (e.g. dapagliflozin, empaglieflozin and ertugliflozin). All eligible medications will be considered as drug classes, and RCTs comparing the same drug class will be excluded, such as comparing different brands of metformin. For example, RCTs of intraclass comparisons of SGLT-2 inhibitors and GLP-1 analogues will be considered meeting the eligibility criteria because of its variable effects on cardiovascular end-point [19]. ADMs were withdrawn, are no longer available, or are not used in clinical practice were not eligible (i.e. albiglutide, rosiglitazone and taspoglutide). The definition of comparisons or control group used for this review is (a) placebo; (b) routine care; or (c) any other ADM (different with intervention drugs). The minimum duration of intervention and follow-up should be at least 3 weeks.

### Information sources and search strategy

We will identify trials through systematic searches of the following bibliographic databases:

Cochrane Library Central Register of Controlled Trials, PubMed, EMBASE, CINAHL, Cochrane Library, Scopus, grey literature (ClinicalTrials.gov, PsycEXTRA, ProQuest Dissertations, Google Scholar and Theses Global). We will also manually search for international trial registries, websites of regulatory agencies (U.S. FDA and Australian Pharmaceutical Benefits Scheme to find registered studies but have not published studies), pharmaceutical companies and critical scientific journals in the field. When there are some unpublished data in some approved projects/studies, we will contact the authors if necessary. We will keep the FDA data separate and the published data so there is a pair of meta-analyses.

To reduce the publication bias because of the selective availability of data, especially in drug research, we will include grey literature (i.e. non-published, internal or non-reviewed articles, repositories) after reviewing the title and abstract accordingly. Additionally, the reference list of identified systematic reviews, NMA and RCTs will also be updated to identify if references or bibliographies include relevant studies that can be included for the review (cross-referencing). We have developed a preliminary search strategy for PubMed (Table S2) through discussions with a medical librarian experienced in conducting systematic database searching (R F, the University Liaison Librarian at the University of Adelaide). These will be adapted for use in other databases.

### Study selection

We will use citation management software (Endnote X9) to manage all studies exported from all the databases. After de-duplication, the pairs of reviewers (MZ, YL, AK, AP, QH, WL, XZ or PH) will screen the titles and abstracts independently using Covidence (https://www.covidence.org). The full-text of nominated studies will be screened at least twice by two different independently reviewers mentioned above based on the pre-defined eligibility criteria using Endnote X9. The process of study screening and selection will be reported according to the PRISMA flow diagram [20] (Table S3).

### Data extraction and management

We will extract data at all time points of intervention for each outcome, then preferentially chose time points reported consistently across studies. Data will be screened and extracted in pairs. A data extraction form covering the information on population, intervention, comparison and outcome measures will be designed based on the guidelines for data extraction and synthesis by the Joanna Briggs Institute [21]. To ensure the reliability of the data extraction process, the data extraction form will be pilot tested by two independent reviewers (MZ and AK). To be specific, we will extract data in the sequence of the form about characteristics of the studies to be included in the current study (including author, publication year, country, sample size, duration of diabetes, patient’s baseline, clinic history, basic treatment, and intervention/treatment duration). Primary outcomes will be HbA1c (≤ 7.0%), time spent with hypoglycaemia (< 70 mg/dl) or hyperglycaemia (≥ 180 mg/dl). Secondary outcomes will be change in weight, blood pressure and related comorbidities (cardiovascular mortality, heart failure events, myocardial infarction and stroke). Continuous variables will be demonstrated as mean values, standard deviations (SD), standard errors, or 95% confidence interval (CI) as and where applicable, whereas categorical variables will be expressed as frequencies and percentages (%), odds ratio (OR) with 95% CIs. We will evaluate duplicate publication, assess all available data simultaneously, maximising the extraction of data for a bias assessment precisely. Authors will be contacted by emails to acquire missing or relevant material of their publications if necessary.

Risk of bias assessment

Two reviewers (MZ and AK) will independently evaluate the quality of each study that meet the inclusion criteria for the systematic review. Conflicts will be resolved by a third author (PH or AP). The latest revised Cochrane’s risk of bias tool will be used for evaluating the quality of RCTs [22]. The following seven constructs will be evaluated as low, moderate, and high risk of bias or unclear risk of bias: random sequence generation, allocation concealment, blinding of personnel and participants, blinding of outcome assessors, incomplete data, selective reporting and other potential risks (Table S4).

### Strength of evidence

The strength of evidence of the NMA will be assessed as follows: risk of bias, inconsistency, indirectness, imprecision (random error) and publication bias based on the Grading of Recommendations Assessment, Development and Evaluation (GRADE) tool [23]. GRADE will be used to evaluate the certainty of evidence. To avoid the limitations of NMA, the following suggested framework for evaluating NMA: (a) the critical role of indirect comparisons; (b) the contributions of each piece of direct evidence to the NMA estimates of effect size; (c) the importance of the transitivity assumption to the validity of NMA; and (d) the possibility of disagreement between direct evidence and indirect evidence [24]. Moreover, two reviewers (QH and YL) will independently evaluate the strength of evidence for each outcome. Any disagreement at this stage will be discussed and resolved by a third reviewer (WL). The summary table of the quality of all included RCTs will be presented following the GRADE principle.

### Data synthesis and statistical analysis

If the quantitative units are expressed differently analysis will be reported using standardised mean differences (SMD) or mean differences if the units are similar with respective 95% CIs. Categorical variables will be analysed and reported using odds ratio with its respective 95% CIs. To explore the consistency of the direct and indirect evidence, we will compare the results by Bayesian NMA with the results of Bayesian meta-analysis with in the same analytical framework.

In this review, the amount of heterogeneity will be assumed as the same or similar settings for all treatment comparisons. The transitivity assumption will be evaluated by comparing the distribution of clinical and methodological variables that could be considered as effect modifiers [25]. The consistency will be evaluated by the design-by treatment test, and by separating direct from indirect evidence [26]. Stata 16.1 will be used to analyse data. Mean difference with 95% CIs will be reported for similar units and standardised mean difference with 95% CIs for varying units.

We will combine the published and unpublished data to form a network and will be included in the meta-analysis. No data will be excluded based on publication status. Later, we will perform a sensitivity analysis on the published data only. Sensitivity analysis will be performed by adding or deleting studies to test the robustness of the choices made in the published RCTs if studies contributed to high heterogeneity. The heterogeneity (both clinical and statistical heterogeneity) will be described via reporting differences in the study design and the characteristics of the study population, which will be deduced by the *I*^2^ statistics [27]. In this sense, we will use *I*^2^ to evaluate statistical heterogeneity (*I*^2^ ≥ 50% is considered as heterogeneous) [28]. We will adopt the random-effect model for meta-analysis if appropriate, such as over five studies are included [29]. If the review includes sufficient RCTs with low variability among those studies, the following analysis of subgroups will be conducted: (a) efficacy of traditional classes of oral ADMs; (b) efficacy of new oral ADMs. A funnel plot will be plotted to evaluate publication bias if there are more than 10 RCTs included. In addition, Eggers’ regression test will be used to statistically evaluate the asymmetry of the funnel plot. Additionally, the review will form the following meta-regression analysis that will be conducted on whether baseline information such as HbA1c, gender, age and types of drugs affect the impact of CGM on HbA1c levels if heterogeneity is identified. Regarding measurement inconsistency, a Bayesian framework and Markov Chain Monte Carlo (MCMC) [30] will be performed by WinBUGs (version 1.4.3) using MCMC packages. MCMC will be used to estimate the posterior distribution for treatment comparison. The frequentist meta-analysis and meta-regression analysis will be conducted in Stata 16.1.

## Discussion

Accurate regulation of blood glucose remains the top priority in the management of T2D. An NMA in this area is required to analyse the effectiveness of glucose control and hyperglycaemia and hypoglycaemia events detected by CGMs among adults with T2D. Besides, it will also analyse the subgroups by comparing the direct and indirect evidence among various ADM types to help further improve diabetes medication management. Our results will be used to inform healthcare providers, policymakers, T2D patients and family members of the relative effectiveness of traditional and new classes of oral ADMs. We will implement rigorous and evidence-based knowledge and translation strategies to ensure that our results will reach key stakeholders such as pharmacists, doctors, and patients.

This review and NMA will have some strengths. To improve the quality of the review, a pre-defined method will be used based on the Cochrane Handbook for Systematic Reviews of Interventions [25]. Moreover, the strength of evidence will be assessed based on GRADE [31]. In addition, direct and indirect evidence from different drug interventions or placebos will be assessed jointly and individually, so that the review will provide sufficient evidence to compare the efficacy of different anti-diabetic drugs through CGM as an assessment tool. Therefore, risks of random error and systematic error of the review will be avoided [32]. Moreover, this network meta-analysis will take place within the multi-disciplinary review team with the expertise in epidemiology, clinical nursing, biostatistics, public health, dietetics and primary care.

A potential limitation or bias of this systematic review could be that we will only include studies that evaluate the effectiveness and safety of ADM assessed by CGM because our research interests focus on glucose fluctuations, especially hypoglycaemia and hyperglycaemia. The review is anticipated to provide up to date evidence for further studies and clinical practice regarding effectiveness and glucose control regarding hypoglycaemia and hyperglycaemia issues.

## Supplementary Information

**Additional file 1: Table S1.** Preferred Reporting Items for Systematic Review and Meta-Analysis Protocols 2015 (PRISMA-P-2015).

**Additional file 2: Table S2.** Logic Grid and preliminary search strategy for PubMed.

**Additional file 3: Table S3.** PRISMA-2009 Flow Diagram.

**Additional file 4: Table S4.** Classification of randomised trails at low and at high risk of biases.

## Data Availability

All data relevant to the study are included in the article or uploaded as supplementary information.

## Abbreviations

CGM: Continuous glucose monitoring
T2D: Type 2 diabetes
ADM: Oral anti-diabetic medications
RCTs: Randomised controlled trials
HbA1c: Glycosylated haemoglobin
DPP-4: Dipeptidyl peptidase-4 inhibitors
SGLT-2: Sodium-glucose co-transporter 2 inhibitors
GLP-1: Glucagon-like peptide 1 agonists
FDA: Food and Drug Administration
NMA: Network meta-analysis
GRADE: Grading of Recommendations Assessment, Development and Evaluation Tool
PROSPERO: International Prospective Register of Systematic Reviews
PRISMA: Preferred Reporting Items for Systematic Reviews and Meta-analyses
PICOS: Population-Intervention-Comparators-Outcomes-Study design
SD: Standard deviation
CI: Confidence interval
MCMC: Markov Chain Monte Carlo
