# Supplementary material for: Changes in glycaemic control of oral anti-diabetic medications assessed by continuous glucose monitors among patients with type 2 diabetes: a protocol of network meta-analysis
Source: Syst Rev. 2022 Jun 2;11:110. doi: 10.1186/s13643-022-01986-5 (PMC9161457; doi:10.1186/s13643-022-01986-5)
Supplement: Supplementary file 2 — Additional file 2: Table S2. Logic Grid and preliminary search strategy for PubMed. [file 13643_2022_1986_MOESM2_ESM.docx]

**Table S2. Logic Grid and preliminary search strategy for PubMed**

***Changes in glycemic control of oral anti-diabetic medications assessed by continuous glucose monitors among patients with type 2 diabetes: A protocol of network meta-analysis of randomised controlled trial***

**Limited to Humans and English; Only terms to be used in the Title & Abstract**

| **Population**  **(Adults with type 2 diabetes)** | **Evaluation (All type of continuous glucose monitoring)** | **Intervention**  **(Anti-diabetic medications)** | **Study** |
| --- | --- | --- | --- |
| **"Diabetes Mellitus, Type 2"[Mesh]**  Diabetes Mellitus, Noninsulin-Dependent  Diabetes Mellitus, Ketosis-Resistant  Diabetes Mellitus, Ketosis Resistant  Ketosis-Resistant Diabetes Mellitus  Diabetes Mellitus, Non Insulin Dependent  Diabetes Mellitus, Non-Insulin-Dependent  Non-Insulin-Dependent Diabetes Mellitus  Diabetes Mellitus, Stable  Stable Diabetes Mellitus  Diabetes Mellitus, Type II  NIDDM  Diabetes Mellitus, Noninsulin Dependent  Diabetes Mellitus, Maturity-Onset  Diabetes Mellitus, Maturity Onset  Maturity-Onset Diabetes Mellitus  Maturity Onset Diabetes Mellitus  MODY  Diabetes Mellitus, Slow-Onset  Diabetes Mellitus, Slow Onset  Slow-Onset Diabetes Mellitus  Type 2 Diabetes Mellitus  Noninsulin-Dependent Diabetes Mellitus  Noninsulin Dependent Diabetes Mellitus  Maturity-Onset Diabetes  Diabetes, Maturity-Onset  Maturity Onset Diabetes  Type 2 Diabetes  Diabetes, Type 2  Diabetes Mellitus, Adult-Onset  Adult-Onset Diabetes Mellitus  Diabetes Mellitus, Adult Onset | Continuous glucose monitoring system*  continuous glucose monitor∗  continuous glucose sens∗  continuous glucose device∗  continuous blood sugar monitor∗  continuous blood sugar sens∗  continuous blood device∗  continuous subcutaneous glucose monitor∗  continuous subcutaneous glucose sens∗  CGM∗  real-time CGM∗  real time CGM ∗  rt-CGM∗  rt CGM∗  fash glucose monitor∗  FGM∗  sensor-augmented insulin pump  sensor augmented insulin pump  SAP  iPro*  FreeStyle Libre*  HiBell*  Dexcom*  MiniMed*  Medtronic*  Guardian Connect CGM*  Senseonics Eversense*  GlucoTrack* | metformin  sulfonylurea  dipeptidyl peptidase-4 inhibitors  DPP-4 inhibitors  DPP-4  sodium-glucose co-transporter type 2 inhibitors  SGLT-2 inhibitors  SGLT-2  thiazolidinediones  TZD  glucagon-like peptide 1 agonists  GLP-1 | (((((((randomized controlled trial[Publication Type]) OR controlled clinical trial[Publication Type]) OR randomized[Title/Abstract]) OR placebo[Title/Abstract]) OR clinical trials as topic[MeSH Major Topic]) OR randomly[Title/Abstract]) OR trial[Title]) |

**Preliminary search strategy for PubMed**

| **#** | **Searches** |
| --- | --- |
| 1 | "Diabetes Mellitus, Type 2"[Mesh] |
| 2 | (((((((((((((((((((((((((((((((((((Diabetes Mellitus, Noninsulin-Dependent) OR Diabetes Mellitus, Ketosis-Resistant) OR Diabetes Mellitus, Ketosis Resistant) OR Ketosis-Resistant Diabetes Mellitus) OR Diabetes Mellitus, Non Insulin Dependent) OR Diabetes Mellitus, Non-Insulin-Dependent) OR Non-Insulin-Dependent Diabetes Mellitus) OR Diabetes Mellitus, Stable) OR Stable Diabetes Mellitus) OR Diabetes Mellitus, Type II) OR NIDDM) OR Diabetes Mellitus, Noninsulin Dependent) OR Diabetes Mellitus, Maturity-Onset) OR Diabetes Mellitus, Maturity Onset) OR Maturity-Onset Diabetes Mellitus) OR Maturity Onset Diabetes Mellitus) OR MODY) OR Diabetes Mellitus, Slow-Onset) OR Diabetes Mellitus, Slow Onset) OR Slow-Onset Diabetes Mellitus) OR Type 2 Diabetes Mellitus) OR Noninsulin-Dependent Diabetes Mellitus) OR Noninsulin Dependent Diabetes Mellitus) OR Maturity-Onset Diabetes) OR Diabetes, Maturity-Onset) OR Maturity Onset Diabetes) OR Type 2 Diabetes) OR Diabetes, Type 2) OR Diabetes Mellitus, Adult-Onset) OR Adult-Onset Diabetes Mellitus) OR Diabetes Mellitus, Adult Onset)) |
| 3 | 1 OR 2 |
| 4 | (((((((((((iPro*) OR freestyle Libre*) OR HiBell*) OR Dexcom*) OR MiniMed*) OR Medtronic*) OR guardian connect CGM*) OR senseonics Eversense*) OR GlucoTrack*)) |
| 5 | (((((((((((((((((((continuous glucose monitoring system*) OR continuous glucose monitor *) OR continuous glucose sens *) OR continuous glucose device *) OR continuous blood sugar monitor *) OR continuous blood sugar sens *) OR continuous blood device *) OR continuous subcutaneous glucose monitor *) OR continuous subcutaneous glucose sens *) OR CGM *) OR real-time cgm *) OR real time cgm *) OR rt-cgm *) OR rt cgm *) OR flash glucose monitor *) OR FGM *) OR sensor-augmented insulin pump) OR sensor augmented insulin pump) OR SAP))) |
| 6 | 4 OR 5 |
| 7 | (((((((randomized controlled trial[Publication Type]) OR controlled clinical trial[Publication Type]) OR randomized[Title/Abstract]) OR placebo[Title/Abstract]) OR clinical trials as topic[MeSH Major Topic]) OR randomly[Title/Abstract]) OR trial[Title]) |
| 8 | mixed model*[TIAB] OR mixed design*[TIAB] OR multiple method*[TIAB] OR multimethod*[TIAB] OR triangulat*[TIAB] |
| 9 | 7 OR 8 |
| 10 | (((((((((((metformin) OR (sulfonylurea)) OR (dipeptidyl peptidase-4 inhibitors)) OR (DPP-4 inhibitors)) OR (sodium-glucose co-transporter type 2 inhibitors)) OR (SGLT-2 inhibitors)) OR (thiazolidinediones)) OR (TZD)) OR (glucagon-like peptide 1 agonists)) OR (GLP-1)) OR (DPP-4)) OR (SGLT-2) |
| 9 | 3 AND 6 AND 9 AND 10 |
| 10 | Filters: Full text; Humans |
| 11 | AND adult* |
