# Supplementary material for: Changes in glycaemic control of oral anti-diabetic medications assessed by continuous glucose monitors among patients with type 2 diabetes: a protocol of network meta-analysis
Source: Syst Rev. 2022 Jun 2;11:110. doi: 10.1186/s13643-022-01986-5 (PMC9161457; doi:10.1186/s13643-022-01986-5)
Supplement: Supplementary file 3 — Additional file 3: Table S3. PRISMA-2009 Flow Diagram. [file 13643_2022_1986_MOESM3_ESM.doc]

**Table S3. PRISMA-2009 Flow Diagram**

**Screening**

**Included**

**Eligibility**

**Identification**

Records identified through database searching
(n = )

Additional records identified through other sources
(n = )

Records after duplicates removed
(n = )

Records screened
(n = )

Records excluded
(n = )

Full-text articles assessed for eligibility
(n = )

Full-text articles excluded, with reasons
(n = )

Studies included in quantitative synthesis
(n = )

Grey literature

(n = )

Cochrane Library

(n = )

PubMed

(n = )

Scopus

(n = )

EMBASE

(n = )

CINAHL

(n = )

PsycINFO (n = )
