# Supplementary material for: Changes in glycaemic control of oral anti-diabetic medications assessed by continuous glucose monitors among patients with type 2 diabetes: a protocol of network meta-analysis
Source: Syst Rev. 2022 Jun 2;11:110. doi: 10.1186/s13643-022-01986-5 (PMC9161457; doi:10.1186/s13643-022-01986-5)
Supplement: Supplementary file 4 — Additional file 4: Table S4. Classification of randomised trails at low and at high risk of biases. [file 13643_2022_1986_MOESM4_ESM.docx]

**Table S4. Classification of randomised trails at low and at high risk of biases**

|  | **High risk of bias** | **Unclear risk of bias** | **Low risk of bias** |
| --- | --- | --- | --- |
| **Random sequence generation** | If the allocation sequence is not randomised or only quasi-randomised. These trials will be excluded. | If the method of randomisation was not specified, but the trial was still presented as being randomized | If sequence generation was achieved using computer random number generator or a random number table. Drawing lots, tossing a coin, shuffling cards, and throwing dice were also considered adequate if performed by an independent adjudicator |
| **Allocation concealment** | If the allocation sequence was familiar to the investigators who assigned participants. | If the trial was classified as randomized but the allocation concealment process was not described. | If the allocation of patients was performed by a central independent unit, on-site locked computer or identical-looking numbered sealed envelopes |
| **Blinding of participants and treatment providers** | If blinding of participants and the treatment providers was not performed. | If the procedure of blinding was insufficiently described. | If the participants and the treatment providers were blinded to intervention allocation and this was described. |
| **Blinding of outcome assessment** | If no blinding or incomplete blinding of outcome assessors was performed. | If it was not mentioned if the outcome assessors in the trial were blinded or the extent of blinding was insufficiently described. | If it was mentioned that outcome assessors were blinded and this was described. |
| **Incomplete outcome data** | If the results were likely to be biased due to missing data either because the pattern of drop-outs could be described as being different in the two intervention groups or the trial used improper methods in dealing with the missing data (e.g. last observation carried forward). | If there was insufficient information to assess whether missing data were likely to induce bias on the results. | If missing data were unlikely to make treatment effects depart from plausible values. This could be either (1) there were no drop-outs or withdrawals for all outcomes, or (2) the numbers and reasons for the withdrawals and drop-outs for all outcomes were clearly stated and could be described as being similar to both groups. Generally, the trial is judged as at a low risk of bias due to incomplete outcome data if drop-outs are less than 5%. However, the 5% cut-off is not definitive. |
| **Selective outcome reporting** | If the outcomes in the protocol were not reported. | If no protocol was published and the outcome of serious adverse events were not reported. | If a protocol was published before or at the time the trial was begun and the outcomes specified in the protocol were reported on. If there is no protocol or the protocol was published after the trial has begun, reporting of serious adverse events will grant the trial a grade of low risk of bias. |
| **Other potential risks of bias** | If there are other factors in the trial that could put it at risk of bias (for example, authors conducted trials on the same topic, for- profit bias, etc.). | If the trial may or may not be free of other components that could put it at risk of bias. | If the trial appears to be free of other components (for example, academic bias or for-profit bias) that could put it at risk of bias. |
| **Overall risk of bias** | If any of the bias risk domains described in the above are classified as ‘unclear’ or ‘high risk of bias’. | N/A | Only if all of the bias domains described in the above paragraphs are classified as ‘low risk of bias’. |

Notes: if the bias is between high and low risk, it can be identified as moderate risk of bias after the discussion by the review team.
